# Clinical Approach to Supranuclear Brainstem Saccadic Gaze Palsies

**DOI:** 10.3389/fneur.2017.00429

**Published:** 2017-08-23

**Authors:** Alexandra Lloyd-Smith Sequeira, John-Ross Rizzo, Janet C. Rucker

**Affiliations:** ^1^Department of Neurology, New York University School of Medicine, New York, NY, United States; ^2^Department of Physical Medicine and Rehabilitation, New York University School of Medicine, New York, NY, United States; ^3^Department of Ophthalmology, New York University School of Medicine, New York, NY, United States

**Keywords:** supranuclear, saccades, burst neuron, progressive supranuclear palsy, slow saccades

## Abstract

Failure of brainstem supranuclear centers for saccadic eye movements results in the clinical presence of a brainstem-mediated supranuclear saccadic gaze palsy (SGP), which is manifested as slowing of saccades with or without range of motion limitation of eye movements and as loss of quick phases of optokinetic nystagmus. Limitation in the range of motion of eye movements is typically worse with saccades than with smooth pursuit and is overcome with vestibular–ocular reflexive eye movements. The differential diagnosis of SGPs is broad, although acute-onset SGP is most often from brainstem infarction and chronic vertical SGP is most commonly caused by the neurodegenerative condition progressive supranuclear palsy. In this review, we discuss the brainstem anatomy and physiology of the brainstem saccade-generating network; we discuss the clinical features of SGPs, with an emphasis on insights from quantitative ocular motor recordings; and we consider the broad differential diagnosis of SGPs.

The goal of the ocular motor system is achievement of single, clear vision via maintenance of an object of visual interest on the fovea, the specialized retinal region with the greatest photoreceptor density. To achieve this, several functional classes of eye movements exist, including saccades, smooth pursuit, optokinetic nystagmus (OKN), vestibular reflexes, and vergence—each served by distinct cortical, brainstem, and cerebellar supranuclear networks. Failure of brainstem supranuclear saccade centers results in a brainstem-mediated supranuclear gaze palsy, which we refer to as a saccadic gaze palsy (SGP). We review the anatomy and physiology of brainstem immediate premotor saccade-initiating neurons and discuss SGP clinical features and its differential diagnosis. Comprehensive coverage of networks involved in saccade generation and termination is beyond the scope of this article but can be reviewed elsewhere (1).

## Brainstem Anatomy and Physiology of Saccadic Generators

**Figure 1:**
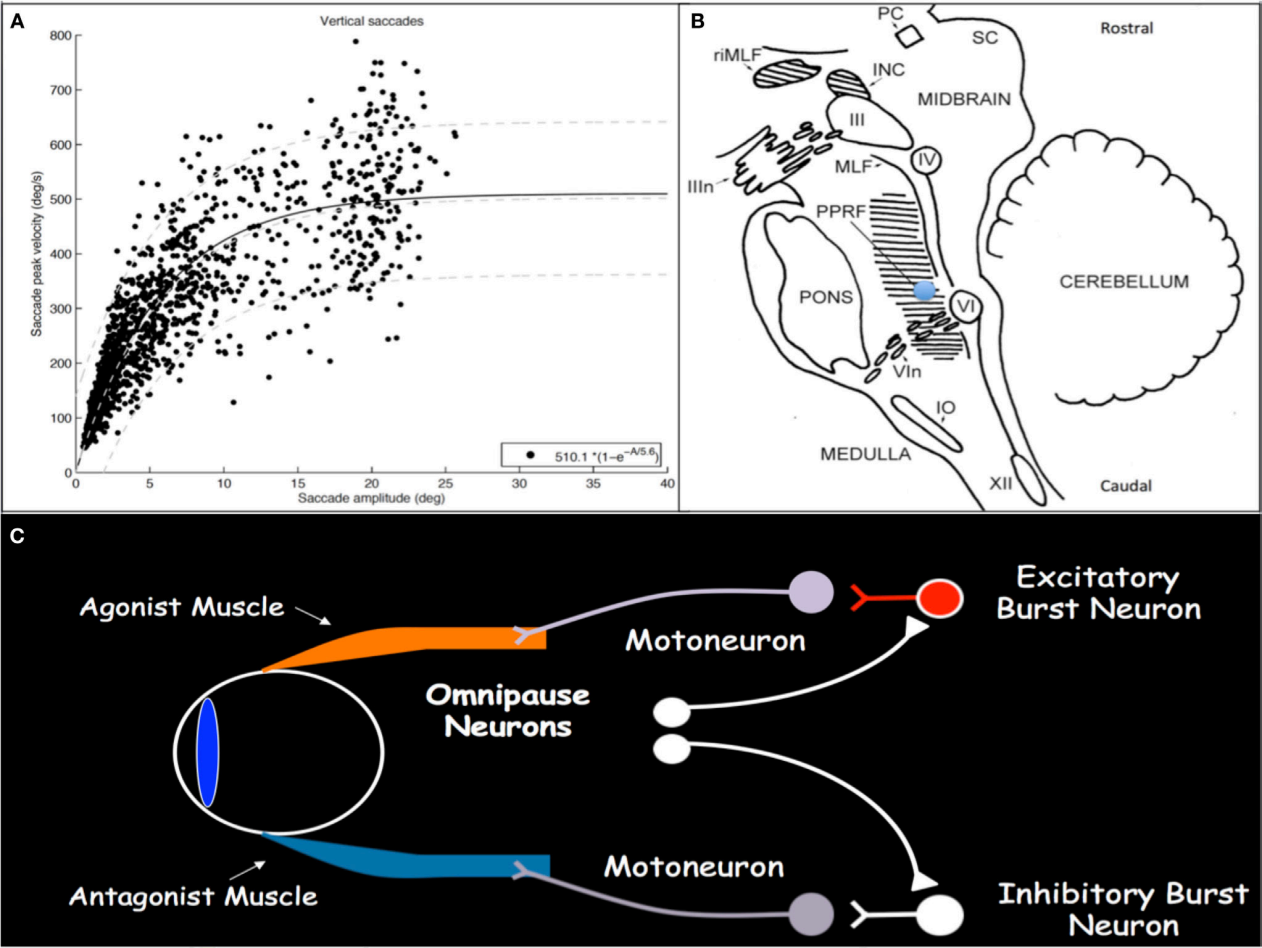
**(A)** Main sequence plot for vertical saccades, representing the relationships between saccade amplitude and peak velocity, in a cohort of patients with concussion demonstrating normal saccadic velocities. As saccade amplitude increases, peak velocity increases in an asymptotic distribution. Light gray lines represent the 5th, 50th, and 95th percentiles, respectively, from bottom to top, in healthy disease-free controls. **(B)** Sagittal brainstem drawing showing the localization of ocular motor-related nuclei. Supranuclear burst neurons for vertical saccades are located in the midbrain in the RIMLF. The shaded region in the pons represents the PPRF, containing supranuclear burst neurons for horizontal saccades. Excitatory burst neurons are located in the region of the blue circle. Abbreviations: PC, posterior commissure; RIMLF, rostral interstitial medial longitudinal fasciculus; INC, interstitial nucleus of Cajal; SC, superior colliculus; IIIn, oculomotor nerve fascicle; III, oculomotor nucleus; IV, trochlear nucleus; MLF, medial longitudinal fasciculus; PPRF, paramedian pontine reticular formation; VI, abducens nucleus; VIn, abducens nerve rootlets; IO, inferior olive; XII, hypoglossal nerve. Drawing based on Buttner and Buttner-Ennever (6). **(C)** Schematic drawing of excitatory and inhibitory burst neurons, omnipause neurons, and their connections with agonist and antagonist extraocular muscles.

Saccades are rapid eye movements with which gaze is shifted to direct the fovea to objects of visual interest and explore the visual world (2, 3). Saccadic eye movements range from intentional volitional movements to reflexive involuntary movements to the quick phases of OKN. Assessment for loss of the latter is particularly helpful in early SGP detection. Saccades must be brief, most with duration less than 100 ms; they must be accurate to land the fovea on target; and they have very high velocities. Duration and velocity are a function of saccade size, with relationships referred to as and characterized by the saccade main sequences (Figure 1A) (4, 5). Peak velocity increases linearly for saccades smaller than 20°; however, for saccades larger than 20°, peak velocity saturates around 500°/s. These main sequence relationships allow for establishment of normal saccadic velocity ranges and are particularly helpful in the context of SGP, with which saccadic velocities become slow.

Execution of a saccade requires an initial burst of neuronal discharge, called the pulse, by excitatory burst neurons (EBNs) in the brainstem reticular formation to agonist ocular motoneurons (Figure 1C) (7, 8). This pulse results in vigorous contraction of the agonist muscle. The pulse is then gradually transitioned to a new tonic step innervation that maintains the eyes in the new position and is generated by neural integrators that include the medullary medial vestibular nucleus and nucleus prepositus hypoglossi for horizontal movements and the interstitial nucleus of Cajal (INC) for vertical and torsional movements (1). Simultaneous with EBN pulse firing, inhibitory burst neurons in the medullary reticular formation, caudal to the abducens nucleus, relax the antagonist muscle (9, 10). When no saccade is being generated, burst neurons are tonically inhibited by glycinergic omnipause neurons in the caudal pontine nucleus raphe interpositus (11–13).

Excitatory burst neurons for horizontal saccades are located in the paramedian pontine reticular formation (PPRF) in the pons just rostral to the abducens nucleus and, for vertical and torsional saccades, in the rostral interstitial medial longitudinal fasciculus (RIMLF) rostral to the oculomotor nucleus in the mesencephalic reticular formation, although a few are also located in the INC just caudal to the RIMLF (Figure 1B) (14–16). Horizontal EBNs project to ipsilateral motoneurons for ipsilateral saccades. For vertical saccades, the projection is to yoked muscle pairs (e.g., inferior rectus and superior oblique muscles for downward saccades), with bilateral projection to elevator muscles and unilateral projection to depressor muscles (17–19). RIMLF EBNs promote rapid torsional movements only ipsilaterally (e.g., the right RIMLF causes rotation of the top poles of the eyes toward the right ear) (20, 21).

## Clinical Features of SGPs

Exam detection of SGP requires assessment not only of the static range of ocular motility, but also of dynamic eye movements in three planes. Saccades, smooth pursuit, vestibular–ocular reflexes, and OKN should be assessed horizontally and vertically (Part 1 of Video S1 in Supplementary Material). Saccades are tested by having the patient make rapid jumps with their eyes between two stationary visual targets, while noting ease of initiation, speed, accuracy, and direction or trajectory. A general “rule-of-thumb” regarding saccade speed is that one should not be able to watch the eye move through the full trajectory. If the eye can be visualized through the full trajectory of motion, the saccade is too slow. Smooth pursuit is tested by having the patient follow a slowly moving target, while observing for corrective saccades. Vestibular–ocular reflexes are tested by passive head movement while the patient fixates a central target, noting the smoothness and range of eye movements. OKN is examined by moving a striped drum or tape in front of the patient, while observing for slow following movements of the eyes and corrective saccadic quick phases. Torsional quick phases are assessed by rolling the head back and forth, bringing each ear toward each shoulder (Part 4 of Video S1 in Supplementary Material).

Saccadic gaze palsy will result in slowing of saccades horizontally or vertically (or both) with or without range limitations. Saccade slowing in isolation is evidence of SGP, even with full eye movement range. It is important to note that isolated mildly impaired eye elevation is not sufficient to diagnose SGP, as this may be seen in healthy elderly individuals as a result mechanical orbital changes (22). Some patients with selective slowing of horizontal or vertical saccades will demonstrate a curved trajectory with saccade testing (Part 3 of Video S1 in Supplementary Material). For example, in vertical SGP attempted vertical saccades may display a lateral curved trajectory, so called “round-the-house” saccades (23–26). Range deficits may also be seen during smooth pursuit, although will tend to be more severe with saccades and should be fully overcome with vestibular–ocular reflexes (Part 2 of Video S1 in Supplementary Material). This establishes the deficit as supranuclear, as vestibular–ocular supranuclear commands travel separately from saccade commands. The classic finding of OKN with SGP is loss of quick phases with a slow tonic deviation of the eyes in the direction of stimulus motion.

Pathology affecting PPRF causes horizontal SGP (Part 3 of Video S1 in Supplementary Material) (27). A unilateral lesion will cause ipsilateral conjugate gaze palsy (28). A bilateral lesion will cause horizontal conjugate gaze impairment and slowing of vertical saccades (29–32). Pathology affecting RIMLF causes vertical SGP (Part 2 of Video S1 in Supplementary Material) and affects torsional quick phases. Each RIMLF projects bilaterally to motoneurons for elevation but only unilaterally for depression, thus, RIMLF lesions theoretically have a more profound effect on downgaze. Bilateral lesions tend to cause loss of downward or all vertical saccades and abolish all torsional quick phases. The effects of unilateral lesions are less well understood. In theory, a unilateral lesion should abolish ipsilesional torsional quick phases (Part 4 of Video S1 in Supplementary Material) (33) and mildly affect downward saccades; however published reports describe more extensive deficits (34). It is likely that other structures, such as the INC, were simultaneously involved in these cases. Monocular vertical SGP is more difficult to understand, but is occasionally seen (35). A specific condition called double elevator palsy results in impairment of both elevator muscles (superior rectus and inferior oblique) in one eye. It is unclear if the lesion is supranuclear or in the oculomotor nucleus or fascicle (36, 37). A specific upgaze SGP occurs in the dorsal midbrain syndrome (e.g., Parinaud’s syndrome) and is accompanied by convergence-retraction nystagmus, Collier’s sign of eyelid retraction, and pupillary light-near dissociation. The SGP is likely due to involvement of projecting fibers from the INC. Classic etiologies include pineal gland neoplasms and hydrocephalus.

Patients with SGP may be visually asymptomatic, due to the symmetric nature of the deficits and lack of ocular misalignment. Diplopia and blurred vision occur more frequently when the deficit has acute onset, such as with infarction.

## Differential Diagnosis of SGP

Although SGP is generally very localizing, it is not pathognomonic for an EBN lesion, as saccade slowing due to dysfunction of the cerebral hemispheres, superior colliculus, and cerebellum has been reported (38–40). The differential diagnosis of brainstem SGP is quite broad, and detailed neurological evaluation with attention to associated symptoms and signs will streamline diagnostic testing and facilitate accurate diagnosis. We consider the differential in mechanistic categories, not comprehensively, but with focus on the most common and recently discovered etiologies.

### Vascular

Acute-onset vertical SGP is typically due to midbrain infarction. The RIMLF is supplied by the posterior thalamo-subthalamic paramedian artery, originating from the posterior cerebral artery. A single perforating artery, the artery of Percheron, supplies both RIMLF in 20% of the population, making bilateral lesions possible from a single vessel infarct (41–43). Unilateral midbrain infarction may also cause bilateral SGP. Acute-onset horizontal SGP is typically due to pontine infarction.

In 1986, horizontal and vertical SGP following cardiac surgery was described (31). Neuropathology revealed pontine and PPRF neuronal necrosis with axonal loss and astrocytosis. Similar cases have since been described (44–49), although further pathology failed to reveal brainstem abnormalities (50), leaving injury localization and mechanism in question. Ischemic injury is considered most likely, given the temporal relationship with cardiac surgery. A recent theory proposes injury to perineural nets that surround brainstem burst neurons (51, 52).

### Neurodegenerative

PSP is a neurodegenerative tauopathy. Its classic form, Richardson syndrome (53), is characterized by early falls, symmetric akinetic parkinsonism with lack of levodopa responsiveness, cognitive impairment, pseudobulbar palsy, and dysphagia. Vertical SGP is the defining characteristic, manifested early as loss of OKN quick phases (54). Excessive square wave jerks are typically present. Selective downgaze impairment is often thought representative; however, slowing of both upward and downward saccades is common and, in a cohort of 30 patients, limitation of upward range was more common (47%) than limitation of both upward and downward-range (30%), and both of the former were more common than selective downward-range limitation (23%) (55). With disease progression, horizontal saccades become affected and complete ophthalmoplegia may occur.

Additional PSP variants include, but are not limited to, corticobasal syndrome and PSP parkinsonism (53, 56), with which eye movement involvement may be subtle or occur late. While SGP with parkinsonism is highly suggestive of PSP; it is not pathognomonic, as SGP may occur in other parkinsonian conditions. In an autopsy series of 27 patients with parkinsonism and supranuclear gaze palsy, pathology was consistent with PSP in 9, Parkinson disease (PD) in 10, corticobasal degeneration in 2, multiple system atrophy in 2, Creutzfeldt–Jakob disease in 1, and Huntington disease in 1 (57). The difficulty in interpreting this study lies in the lack of details regarding eye movement features, making it impossible to differentiate between cortically mediated ocular motor apraxia and brainstem SGP. Those with PD had pathologic changes in the cortex but not in the brainstem and were unlikely to have had brainstem SGP. Saccade slowing may be seen in Lewy body dementia, corticobasal syndrome, and Huntington disease, although it tends to occur late in the disease course (58–61).

Eye movements tend to be relatively spared in amyotrophic lateral sclerosis (ALS), although some patients do exhibit SGP (62–64). In a study of 63 patients with ALS, upgaze was moderately or severely restricted in 13% (65), although it is unclear if this was due to ocular motor apraxia or brainstem SGP. Definite vertical SGP was seen in two patients with RIMLF cell loss at autopsy (62).

### Metabolic/Genetic

Vertical SGP (especially downward) is a key feature of Niemann–Pick type C (NPC), present in 65% (66, 67). NPC is an autosomal-recessive illness caused by mutations in the NPC1 or NPC2 gene, in which cholesterol and lipids accumulate due to a defect in intracellular lipid trafficking. Additional features include gelastic cataplexy and hepatosplenomegaly, although in adult-onset cases, visceromegaly may be absent (68).

Gaucher disease is an autosomal-recessive sphingolipidosis caused by mutations in the GBA gene which decrease glucocerebrosidase activity (69). Gaucher type 3, the subacute neurological form, has later onset and slower progression than types 1 and 2. Horizontal SGP is characteristic, and may be the dominant feature. Additional features include myoclonic epilepsy, cerebellar ataxia, spasticity, or dementia. SGP is slowly progressive (70), and saccades have been utilized as a treatment outcome measure (71, 72).

Spinocerebellar ataxias (SCA) are due to genetic CAG-repeat expansions resulting in protein polyglutamine extension. There is substantial phenotypic overlap between mutations and mild SGP has been reported with many; however horizontal SGP with early, severe slowing of horizontal saccades is characteristic of spinocerebellar ataxia type 2 (73–77), in which saccade dysfunction is correlated with polyglutamine repeats (75).

### Neoplasm

Tumors affecting the pineal gland, including pineal germinoma or teratoma, pineocytoma, pineoblastoma, glioma, or metastasis, can lead to the dorsal midbrain syndrome via compression of the midbrain tectal plate. Rarely, pineal lesions in the elderly mimic PSP (78, 79).

### Paraneoplastic, Autoimmune, and Inflammatory

Ma1 and Ma2 are intracellular proteins expressed in the testes and brain (80). A study of 38 patients with anti-Ma2 encephalitis reported upward greater than downward SGP in 60% (81), some with progression to complete ophthalmoplegia. Additional ocular motor deficits include opsoclonus, ocular flutter, oculogyric crisis, nystagmus (horizontal, horizontal-torsional, and downbeat), and skew deviation. Excessive daytime sleepiness is present in a third of patients. Atypical parkinsonism occurs, thus mimicking PSP (82). It may also mimic Whipple disease with a PSP phenotype and opsoclonus or nystagmus (83).

Anti-glutamic acid decarboxylase (GAD) antibody is associated with many eye movement disorders, including downbeat and periodic alternating nystagmus, ocular flutter and opsoclonus, and ophthalmoplegia with or without stiff person syndrome (SPS) or cerebellar dysfunction (84–87). Early reports of ophthalmoplegia in SPS were attributed to myasthenia gravis and, indeed, patients may have both anti-GAD and acetylcholine receptor antibodies (88). A direct role of anti-GAD and anti-glycine receptor (GlyR) Ab in the pathology of ophthalmoplegia, as well as definitive examination findings compatible with SGP, are reported in the continuum between SPS and progressive encephalomyelitis with rigidity and myoclonus (PERM) (85, 89–93). Anti-GlyR are the hallmark antibodies associated with PERM, which is characterized by brainstem, autonomic, and spinal cord involvement (94). GAD catalyzes the conversion of glutamate to gamma-aminobutyric acid, which has a known role in saccadic premotor control at several levels, including the superior colliculus, vertical inhibitory burst neurons in the INC, and the cerebellum (16, 95, 96).

Anti-IgLON5 antibodies are associated with a novel category of neurological disease, cell surface antibody-associated neurodegeneration, at the border between autoimmune and neurodegenerative disease (97). In an analysis of the largest cohort to date of 22 patients, four syndromic types were identified: (1) sleep disorder predominant (36%), (2) bulbar predominant (27%), (3) PSP-like (25%), and (4) cognitive decline with or without chorea (14%) (98). Despite the presence of vertical SGP in some patients (99), SGP may also be horizontal and the condition differs from PSP in that parkinsonism tends to be absent with anti-IgLON5 Ab and sleep dysfunction is not a prominent PSP feature (98).

Saccadic gaze palsy is rare in demyelinating disease, but may occur as an initial demyelinating event or as an exacerbation in multiple sclerosis (76, 100, 101).

### Prion Disease

Creutzfeldt–Jakob disease (CJD) can cause vertical SGP as an early or late feature and in familial or sporadic cases (102–107). SGP is typically associated with early falls and symmetric akinetic parkinsonism and, thus, mimics PSP. Clinical, radiologic, and laboratory features of CJD may be absent and PSP diagnostic criteria may be met (108), however, the course is rapidly progressive with death ensuing within 1–3 years. Familial CJD due to mutations at prion protein codons 129 and 200 on chromosome 20 has been associated with a PSP-like phenotype (102). The thalamocortical MM2 subtype, responsible for 4% of sporadic CJD cases, may underlie the PSP-like phenotype in sporadic CJD (108).

### Infection

Whipple disease is a chronic infection by gram-positive bacillus *Tropheryma whipplei*. It may mimic PSP, with vertical SGP that may progress to complete ophthalmoplegia. Oculomasticatory myorhythmia is the pathognomonic, although not consistently present, finding consisting of pendular vergence oscillations and concurrent masticatory muscle contractions. Systemic features include gastrointestinal symptoms, weight loss, fever and polyarthralgia. Neurologic involvement includes cerebellar ataxia, tremor, postural instability, dystonia, myoclonus, cognitive deficits, delirium, and seizures.

## Conclusion

Pathophysiologic understanding of brainstem mechanisms that may result in SGP is the first step in accurate identification and localization of this eye movement deficit. Chronic progressive vertical SGP is a core feature of PSP; however the differential diagnosis of SGP is broad and careful consideration must be given to the temporal course and accompanying features to ensure accurate diagnosis.

## Author Contributions

All authors (AL-SS, J-RR, and JR) participated in conception and organization of review, literature search, and all stages of writing—from initial draft to final product.

## Conflict of Interest Statement

The authors declare that the research was conducted in the absence of any commercial or financial relationships that could be construed as a potential conflict of interest.

## Supplementary Material

Video S1 The video contains three separate examinations: part 1. Normal examination techniques of vertical smooth pursuit, saccades, and vestibulo-ocular reflexes; part 2. A patient with a supranuclear saccadic downgaze palsy from a midbrain lesion; and part 3. A patient with spinocerebellar ataxia type 2 with slowed horizontal saccades. Part 1: Normal exam. “Normal smooth pursuit”—note the slowly moving target and smoothness of the full range of vertical pursuit without corrective saccades. “Normal saccades”—note the rapid speed and full range of vertical saccades as saccades are made to examiner command between two vertically separated targets. “Normal vestibulo-ocular reflexes”—note the full range of smooth eye movements as the participant fixates a stationary visual target in front of her while the examiner passively rotates her head in the vertical plane. Part 2. “Impaired downward pursuit”—note the full range and smoothness of excursion from midline to upgaze and back down to midline but the complete inability to pursue below the midline position. “Inability to make downward saccades”—after an initial small upward movement, note the inability of the eyes to make a saccade downward. Halfway through the clip, this patient also demonstrates stimulus-induced blepharospasm and eyelid opening apraxia. “Full downward VOR”—note the full range of downward eye movement elicited by vestibulo-ocular reflexes. This combination of range of motion deficit affecting saccades more than smooth pursuit and overcome by VOR is classic for a brainstem-mediated supranuclear gaze palsy. Part 3. “Slow horizontal saccades”—note the very slow speed of horizontal saccades with retained full range of horizontal movement in the first portion of the clip. Occasional oblique saccades are made, demonstrating the faster vertical movement first with the slower horizontal movement lagging behind. At the end of clip, note the relatively faster speed of vertical saccades. Part 4. “Normal torsional quick phases”—note both fast and slow torsional movements of the eye, invoked by rolling the head back and forth, each ear to each shoulder. “Abnormal unidirectional torsional quick phases”—in this patient with a right rostral interstitial medial longitudinal fasciculus infarction, note that in one direction, only slow torsional movements are seen and fast torsional phases are missing. This occurs when the tops poles of they eyes rotate toward the right shoulder. In the opposite direction, when the top poles of the eyes rotate toward the left shoulder, both fast and slow torsional movements are seen.
